# Optimization of 4D vessel‐selective arterial spin labeling angiography using balanced steady‐state free precession and vessel‐encoding

**DOI:** 10.1002/nbm.3515

**Published:** 2016-04-13

**Authors:** Thomas W. Okell, Peter Schmitt, Xiaoming Bi, Michael A. Chappell, Rob H. N. Tijssen, Fintan Sheerin, Karla L. Miller, Peter Jezzard

**Affiliations:** ^1^ FMRIB CentreNuffield Department of Clinical NeurosciencesUniversity of OxfordOxfordUK; ^2^ MR Application and Workflow DevelopmentSiemens AG, Healthcare SectorErlangenGermany; ^3^ MR R&DSiemens HealthcareLos AngelesCAUSA; ^4^ Institute of Biomedical EngineeringUniversity of OxfordOxfordUK; ^5^ Department of RadiotherapyUniversity Medical Center UtrechtUtrechtThe Netherlands; ^6^ NeuroradiologyOxford University Hospitals NHS TrustOxfordUK

**Keywords:** non‐contrast‐enhanced dynamic (time‐resolved) magnetic resonance angiography, vessel‐selective angiography, vessel‐encoded pseudo‐continuous arterial spin labeling (VEPCASL), balanced steady‐state free precession (bSSFP)

## Abstract

Vessel‐selective dynamic angiograms provide a wealth of useful information about the anatomical and functional status of arteries, including information about collateral flow and blood supply to lesions. Conventional x‐ray techniques are invasive and carry some risks to the patient, so non‐invasive alternatives are desirable. Previously, non‐contrast dynamic MRI angiograms based on arterial spin labeling (ASL) have been demonstrated using both spoiled gradient echo (SPGR) and balanced steady‐state free precession (bSSFP) readout modules, but no direct comparison has been made, and bSSFP optimization over a long readout period has not been fully explored. In this study bSSFP and SPGR are theoretically and experimentally compared for dynamic ASL angiography. Unlike SPGR, bSSFP was found to have a very low ASL signal attenuation rate, even when a relatively large flip angle and short repetition time were used, leading to a threefold improvement in the measured signal‐to‐noise ratio (SNR) efficiency compared with SPGR. For vessel‐selective applications, SNR efficiency can be further improved over single‐artery labeling methods by using a vessel‐encoded pseudo‐continuous ASL (VEPCASL) approach. The combination of a VEPCASL preparation with a time‐resolved bSSFP readout allowed the generation of four‐dimensional (4D; time‐resolved three‐dimensional, 3D) vessel‐selective cerebral angiograms in healthy volunteers with 59 ms temporal resolution. Good quality 4D angiograms were obtained in all subjects, providing comparable structural information to 3D time‐of‐flight images, as well as dynamic information and vessel selectivity, which was shown to be high. A rapid 1.5 min dynamic two‐dimensional version of the sequence yielded similar image features and would be suitable for a busy clinical protocol. Preliminary experiments with bSSFP that included the extracranial vessels showed signal loss in regions of poor magnetic field homogeneity. However, for intracranial vessel‐selective angiography, the proposed bSSFP VEPCASL sequence is highly SNR efficient and could provide useful information in a range of cerebrovascular diseases. © 2016 The Authors. NMR in Biomedicine published by John Wiley & Sons Ltd.

**Abbreviations used**
*2D*/*3D*/*4D*: *two*/*three*/*four dimensional*
*ACA*: *anterior cerebral artery*
*ASL*: *arterial spin labeling*
*bSSFP*: *balanced steady‐state free precession*
*FSL*: *FMRIB Software Library*
*GRAPPA*: *generalized autocalibrating partially parallel acquisitions*
(*R*/*L*)*ICA*: (*right*/*left*) *internal carotid artery*
*MCA*: *middle cerebral artery*
*MIP*: *maximum intensity projection*
*PCA*: *posterior cerebral artery*
*PCASL*: *pseudo‐continuous arterial spin labeling*
*ROI*: *region of interest*
*SNR*: *signal‐to‐noise ratio*
*SPGR*: *spoiled gradient echo*
*TOF*: *time of flight*
(*R*/*L*)*VA*: (*right*/*left*) *vertebral artery*
*VEPCASL*: *vessel‐encoded pseudo‐continuous arterial spin labeling*

## Introduction

Vessel‐selective angiography is important for a range of clinical applications such as assessment of collateral flow, which is crucial for maintaining blood supply to the brain during a stroke 1 or in response to atherosclerotic disease, and to aid in therapeutic planning for patients with stenoses or lesions, such as the endovascular embolization of arteriovenous malformations. Traditional x‐ray techniques are invasive and expensive, and carry significant risks to the patient 2, motivating the development of non‐invasive alternatives.

Arterial spin labeling (ASL) 3, 4 is capable of generating angiographic contrast through the subtraction of images acquired with and without the prior inversion of blood magnetization flowing into the imaging region 5, 6, 7, 8, negating the need for an exogenous contrast agent. Many recent studies have used spoiled gradient echo (SPGR) readouts, for example References 9, 10, 11, 12, 13, 14, but one drawback of this approach is that SPGR significantly attenuates the ASL contrast: every repetition time (*T*_R_) a fraction (1 – cos *α*) of the remaining longitudinal magnetization is lost 15, where *α* is the excitation flip angle. In order to preserve sufficient signal the flip angle and *T*_R_ used for imaging are therefore highly restricted, particularly when a long readout period is required, limiting the achievable signal‐to‐noise ratio (SNR) and increasing the scan time.

One alternative to SPGR is balanced steady‐state free precession (bSSFP), where the excited transverse magnetization is not spoiled away, but “recycled” for the next *T*_R_ period 16, yielding high SNR efficiency, but also sensitivity to off‐resonance effects. bSSFP has been utilized for both ASL perfusion imaging 17, 18 and dynamic angiography 19, 20, 21, 22, 23, 24. However, no direct comparison of SPGR and bSSFP for ASL angiography has yet been made, and optimization of bSSFP parameters over a long readout period has not yet been fully explored in this context.

For vessel‐selective applications, the ASL preparation can be modified to label blood flowing through an individual artery 12, 14, 25, 26. However, such methods are not SNR efficient where multiple arteries are of interest, since labeled blood signal is confined to a single artery on any given acquisition: for example, to generate four vessel‐selective angiograms of the brain‐feeding arteries, the measured signal ***y*** can be described using the equations of Wong 27 as

(1) $$y=\left(\begin{array}{ccccc} -1 & 0 & 0 & 0 & 1\\ 1 & 0 & 0 & 0 & 1\\ 0 & -1 & 0 & 0 & 1\\ 0 & 1 & 0 & 0 & 1\\ 0 & 0 & -1 & 0 & 1\\ 0 & 0 & 1 & 0 & 1\\ 0 & 0 & 0 & -1 & 1\\ 0 & 0 & 0 & 1 & 1 \end{array}\right)\left(\begin{array}{c} R_{ICA}\\ L_{ICA}\\ R_{VA}\\ L_{VA}\\ S \end{array}\right)$$

where *R*_ICA_, *L*_ICA_, *R*_VA_, *L*_VA_, and *S* represent the blood signals arising from the right and left internal carotid arteries, the right and left vertebral arteries, and static tissue, respectively. The SNR efficiency of this scheme 27 is only 50% relative to non‐selective ASL. In addition, where arteries are not widely separated nearby vessels may be partially labeled 26, leading to some ambiguity in the source of the labeled blood.

In vessel‐encoded pseudo‐continuous arterial spin labeling (VEPCASL) 27, a series of images is acquired with different combinations of arteries labeled or unlabeled in each image. Decoding of such images in post‐processing allows the generation of vessel‐specific contrast with high SNR efficiency, as has been demonstrated previously for ASL angiography 10. For example, using an eight‐cycle scheme 28, and assuming that the arteries can be efficiently encoded, we can write the measured signal as

(2) $$y=\left(\begin{array}{ccccc} -1 & -1 & -1 & -1 & 1\\ 1 & 1 & 1 & 1 & 1\\ -1 & 1 & -1 & 1 & 1\\ 1 & -1 & 1 & -1 & 1\\ -1 & -1 & 1 & 1 & 1\\ 1 & 1 & -1 & -1 & 1\\ -1 & 1 & 1 & -1 & 1\\ 1 & -1 & -1 & 1 & 1 \end{array}\right)\left(\begin{array}{c} R_{ICA}\\ L_{ICA}\\ R_{VA}\\ L_{VA}\\ S \end{array}\right)$$

In this case, the SNR efficiency is 100% relative to standard PCASL, yielding vessel‐selective angiograms with twice the SNR of single‐artery selective methods using the same number of measurements.

In this study we aimed to optimize the SNR efficiency of vessel‐selective ASL dynamic angiography by first comparing bSSFP and SPGR readout methods, both in terms of their theoretical impact on the magnetization and the experimentally achievable SNR efficiency. We then optimized bSSFP imaging parameters to maximize the ASL signal over a long readout period and minimize transient signal oscillations. We combined a time‐resolved bSSFP readout with a VEPCASL preparation to generate highly SNR‐efficient vessel‐selective four‐dimensional (4D; dynamic three‐dimensional, 3D) and very rapidly acquired dynamic two‐dimensional (2D) angiograms. Finally, we report on the high vessel selectivity obtained with this technique, along with the potential for off‐resonance sensitivity for combined intra‐ and extra‐cranial applications.

## Methods

### Simulations

In order to demonstrate the potential benefits of bSSFP for ASL angiography over SPGR, the evolution of initially inverted (“tagged”) and relaxed (“controlled”) magnetization was determined via numerical simulation of the Bloch equations. For simplicity, non‐selective hard RF pulses were simulated and SPGR was assumed to have 100% spoiling efficiency. In addition, the action of the imaging gradients was ignored, since in SPGR the transverse magnetization is spoiled at the end of the *T*_R_, so the action of such gradients will have no impact on subsequent *T*_R_ periods. In bSSFP all imaging gradients are assumed to be perfectly balanced, giving no net effect on the magnetization at the end of each *T*_R_ period. Some residual phase accrual due to the gradients may occur in flowing blood, although this has been shown to be minimal when a linear phase‐encode ordering scheme is used 29, as it is in this study. The ASL signal was calculated as the magnitude of the difference in the transverse magnetization of tagged and controlled blood halfway through the *T*_R_ period. The *T*_1_ and *T*_2_ of blood were assumed to be 1650 ms 30 and 150 ms 31, respectively.

The ASL signal achieved by SPGR and bSSFP in three regimes was simulated (i) using parameters suitable for SPGR (*α* = 8°, *T*_R_ = 12 ms), (ii) using a shorter *T*_R_ to increase the *k*‐space sampling rate with a correspondingly smaller flip angle to give comparable SPGR signal attenuation to the first regime (*α* = 5°, *T*_R_ = 4.2 ms), and (iii) using more demanding imaging parameters to increase the initial signal strength along with rapid *k*‐space sampling at a cost of increased signal attenuation rate (*α* = 20°, *T*_R_ = 4.2 ms).

Note that the SPGR signal attenuation rates in regimes (i) and (ii) were approximately matched to a previously published protocol 10 (*α* = 10°, *T*_R_ = 18 ms) by considering that the signal attenuation due to the imaging RF pulses, *R*, at time *t* after the start of imaging is given by 15

(3) $$R={\left(\cos\alpha \right)}^{t/T_{R}}=e^{t\ln\left(\cos\alpha \right)/T_{R}}$$

Therefore, to achieve the same signal attenuation rate as a protocol with flip angle *α*_1_ and repetition time *T*_R,1_ with a new repetition time, *T*_R,2_, the new flip angle, *α*_2_, should be set to

(4) $$\alpha_{2}=\text{acos}\left\{\exp\left[\ln\left(\cos\alpha_{1}\right)\;T_{R,2}/T_{R,1}\right]\right\}$$

In bSSFP the magnetization is flipped back and forth in alternate *T*_R_ periods (i.e. the RF phase alternates between 0 and *π*), meaning that its angle to the longitudinal axis just after each RF pulse is approximately half the applied flip angle. Therefore, the bSSFP flip angle was set to twice that of the nominal flip angle quoted above to ensure that the transverse magnetization generated by the two methods were approximately equal at early time points.

Another consideration for the use of bSSFP in ASL angiography is that imaging has to occur in the transient regime before the magnetization reaches its steady‐state (at which point the contrast between tag and control images would be zero). At these early time points signal oscillations can occur, which lead to modulation of the signal across *k*‐space and therefore significant artifacts 32. Two common approaches used to reduce these oscillations are (a) the “half‐angle” method, in which an RF pulse with flip angle *α*/2 is played out at a time *T*_R_/2 prior to the first *α* pulse 32, and (b) the “linearly increasing” method, in which a number (*N*_p_) of RF pulses separated by *T*_R_ which have flip angles that linearly increase from *α*/(*N*_p_ + 1) to *αN*_p_/(*N*_p_ + 1) are played out prior to the bSSFP readout 33. The half‐angle method requires only a single pre‐pulse, but in general is less robust to magnetization that is off resonance 33. To assess the efficacy of these two techniques for minimizing oscillations in the ASL signal, both were simulated for magnetization at a range of off‐resonance frequencies using *T*_R_ = 4.2 ms and *α* = 40°. For the linearly increasing method, a range of *N*_p_ values was also tested. Finally, the ASL signal produced at a range of different flip angles was simulated (also for *T*_R_ = 4.2 ms) to determine the optimal value to use for subsequent experiments.

### Scan protocol

**Figure 1:**
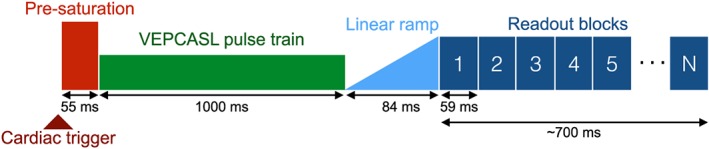
Schematic diagram of the bSSFP VEPCASL dynamic angiography pulse sequence.

The scan protocol consisted of 3D multi‐slab time‐of‐flight (TOF) angiography of the head and neck (0.8 × 0.8 × 1.5 mm^3^, 5 min), which was used to select the VEPCASL labeling plane and establish the location of the two internal carotid arteries (ICAs) and vertebral arteries (VAs) within this plane. This was followed by the 4D VEPCASL bSSFP angiography prototype sequence, as shown in Figure 1, which was triggered to the cardiac cycle via a pulse oximeter.

The pre‐saturation scheme to reduce static tissue signal, the choice of labeling plane, VEPCASL parameters and the eight vessel‐encoding cycles used were identical to those given by Okell *et al*. 28. Briefly, these encodings included two non‐selective, two left–right, two anterior–posterior and two diagonal encodings (as per Equation [2]). The VEPCASL pulse train was played out for 1000 ms followed by 20 RF pulses with linearly increasing flip angles to reduce transient signal oscillations before the segmented bSSFP readout began.

In a similar manner to previous studies 20, 21, the bSSFP readout consisted of a series of blocks, in which the same 14 lines of *k*‐space were acquired in each block. After each VEPCASL preparation a different set of 14 *k*‐space lines was obtained and the process repeated until all necessary lines were acquired. Data from each readout block were used to construct a single image, and with the *T*_R_ set to 4.2 ms this yielded a temporal resolution of 59 ms. The number of readout blocks was set to approximately 12, although this was adjusted for each subject to ensure the VEPCASL preparation and bSSFP readout were complete within two cardiac cycles to minimize dead time before the next cardiac trigger. The voxel size was set to 1 × 1.2 × 4 mm^3^, interpolated to 1 × 1 × 2 mm^3^, with field of view = 220 × 177 × 64 mm^3^, giving a total imaging time of approximately 18 min (the exact value depending on the subject's cardiac cycle). Further imaging parameters are listed in Table 1.

**Table 1:** Sequence parameters for 4D and dynamic 2D VEPCASL angiography. Where only one value is stated this is applicable to both acquisition strategies

Sequence parameter	Value (4D mode | dynamic 2D mode)
VEPCASL pulse train duration	1000 ms
Number of vessel encodings	8
Number of linearly increasing pre‐pulses (*N*_p_)	20
Imaging plane	transverse slab | any orientation
In‐plane field of view	220 × ~177 mm^2^ (subject dependent)
Slab thickness	64 mm | 64–100 mm (orientation dependent)
Slice oversampling	12.5% | –
In‐plane parallel acceleration	GRAPPA factor 2
Voxel size	1.2 × 1.0 × 4.0 mm^3^ | 1.2 × 1.0 × (64–100) mm^3^
Reconstructed voxel size^a^	1.0 × 1.0 × 2.0 mm^3^ | 1.0 × 1.0 × (64–100) mm^3^
Readout partial Fourier	78%
Phase partial Fourier	75%
Slice partial Fourier	75% | –
Readout bandwidth	496 Hz per pixel
Repetition time (*T*_R_)	4.2 ms
Excitations per readout block	14
Temporal resolution	59 ms
Echo time (*T*_E_)	1.8 ms
Excitation flip angle	40°
Number of readout blocks	~12 (cardiac cycle dependent)
Transmit coil	body
Receive coil	12 channel head
Time between VEPCASL preparations	~two cardiac cycles
Total imaging time	~18 min | ~1.5 min

a Via zero‐padding in *k*‐space.

In our previous dynamic 2D SPGR implementation 10, correction for through‐plane motion was not possible, so the acquisition of data for different vessel‐encoding cycles was interleaved to minimize the effect of motion. However, with dynamic 3D data sets retrospective motion correction becomes possible, so in this study all *k*‐space data for each vessel‐encoding cycle were acquired before moving on to the next cycle.

A shorter dynamic 2D bSSFP VEPCASL angiography protocol was also used with parameters identical to those of the 4D sequence (see Table 1), including the same slice thickness (64 mm), but without slice encoding. This yielded images with voxel size 1 × 1.2 mm^2^ in plane and 59 ms temporal resolution, but with the acquisition time reduced to about 1.5 min (cardiac cycle dependent).

### Healthy volunteer experiments

All healthy volunteers were scanned on a 3 T MAGNETOM Trio, a Tim system (Siemens Healthcare, Erlangen, Germany) under a technical development protocol agreed with local ethics and institutional committees. A 12 channel head RF receive coil was used in combination with the body coil for RF transmission.

Five volunteers (four male, one female; age range 25–38) were scanned with the full 4D protocol described above, and four of these were also scanned with the dynamic 2D protocol in the transverse view. A comparison of bSSFP and SPGR dynamic 2D protocols was made in one additional subject (male, 30), with flip angles and *T*_R_ values as listed for the three regimes described in the simulations section. Further imaging parameters for these protocols are given in Supplementary Table 1.

Dynamic 2D VEPCASL bSSFP angiograms were also acquired in coronal and sagittal orientations in another additional subject (male, 25) with the slab thickness increased to 100 mm to encompass the majority of the intra‐ and extra‐cranial arteries.

### Image processing

All images were processed using the FMRIB Software Library (FSL 34) and MATLAB (MathWorks, Natick, MA, USA). Both 4D and dynamic 2D VEPCASL angiography data were motion corrected using MCFLIRT 35. The Brain Extraction Tool 36 was applied to the 3D TOF data to generate a brain mask, which was then linearly registered (using FLIRT 37) and applied to the VEPCASL angiography images to exclude signal from the scalp and eyes.

In order to separate the blood signals arising from each feeding artery from the vessel‐encoded data, a fast maximum *a posteriori* Bayesian version 38 of the general framework for vessel‐encoded analysis 39 was applied to the magnitude images. This method is SNR efficient and compensates for rigid body motion between the vessel localization scan and the vessel‐encoded acquisitions. This analysis results in one vessel‐selective dynamic angiogram for each of the four main brain‐feeding arteries, which can be displayed separately or together using color to represent the vascular origin of the blood signal. Maximum intensity projections (MIPs) of each 4D data set were performed to aid visualization.

Due to the long VEPCASL pulse train (1000 ms), the first acquired time frame shows the vessels filled with labeled blood, with subsequent time frames showing the outflow of this bolus. For a more intuitive visualization, “inflow‐subtraction” was performed, in which each time frame was subtracted from the first. In this way the time at which the bolus leaves the voxel in the original images becomes the time at which the signal appears in the inflow‐subtracted images, giving the appearance of inflow rather than outflow. This method has been previously used by our group 40 and others 11. In practice small residual transient bSSFP artifacts were observed in the first time frame and these would propagate through all subsequent inflow‐subtracted images, so the second time frame was used instead.

Timing information was obtained from both 4D and dynamic 2D data sets using a simple outflow metric: the signal in each voxel was temporally smoothed (Savitzky–Golay filter with polynomial order = 2 and frame size = number of readout blocks – 1) before calculating the time at which it drops to less than 50% of its maximum, as per Okell *et al*. 10. This is approximately equivalent to the time the blood takes to travel from the labeling plane to each voxel if bolus dispersion and signal attenuation due to the RF imaging pulses are minimal.

In order to assess the vessel selectivity of the proposed method, regions of interest (ROIs) were manually defined in the M2 segment of both middle cerebral arteries (MCAs) and in the P2 segment of both posterior cerebral arteries (PCAs) for each subject using the transverse MIPs of the 4D data. In one subject, who had a fetal type circle of Willis on one side, the ipsilateral PCA ROI was drawn in the P1 segment to exclude true ICA signal. To exclude signal outside arteries a vessel mask was created by summing the blood signal across all feeding arteries, determining the 99th percentile of this signal intensity and thresholding the image at 95% of this value, which was found empirically to segment the large arteries well. Within the intersection of each ROI with the vessel mask the fraction of the total signal arising from each feeding artery was calculated. The mean “contamination” signal arising from arteries not expected to contribute to each ROI was then calculated: in the MCA ROIs only the ipsilateral ICA is expected to contribute and in the PCA ROIs only the VAs are expected to contribute (in the case of a typical vascular anatomy).

In order to aid comparison between the bSSFP and SPGR data acquired in one subject, a measure of SNR efficiency was calculated. The “signal” was taken as the mean signal intensity of the ASL angiograms within a vessel mask (as described above), averaged across all feeding arteries and time frames. The “noise” was taken to be the standard deviation of the signal in a 10 × 10 pixel background ROI positioned in the posterior of the brain, away from any arteries, across all feeding arteries and time frames. The ratio of the signal to the noise was then divided by the square root of the acquisition time (in minutes) to yield the SNR efficiency.

Finally, a qualitative comparison of arterial visualization was performed on both the 3D TOF and the 4D VEPCASL angiography data by an experienced neuroradiologist (F.S.) using a scoring system similar to that of Wu *et al*. 41: 0 – not visible/non‐diagnostic; 1 – poor (some structure visible but not clearly defined); 2 – good (diagnostic, clear vessels); 3 – excellent (very clearly delineated). All the major cerebral arterial segments were scored, including the first and second segments of the ACAs, MCAs and PCAs, along with the distal MCA segments (defined as any MCA branches beyond M2). The reader was blind to the subject numbers and data were presented in a randomized order to prevent potential bias. A nonparametric paired Wilcoxon signed rank test was performed to determine whether differences in TOF and VEPCASL angiography scores within each vessel segment were significant.

## Results

**Figure 2:**
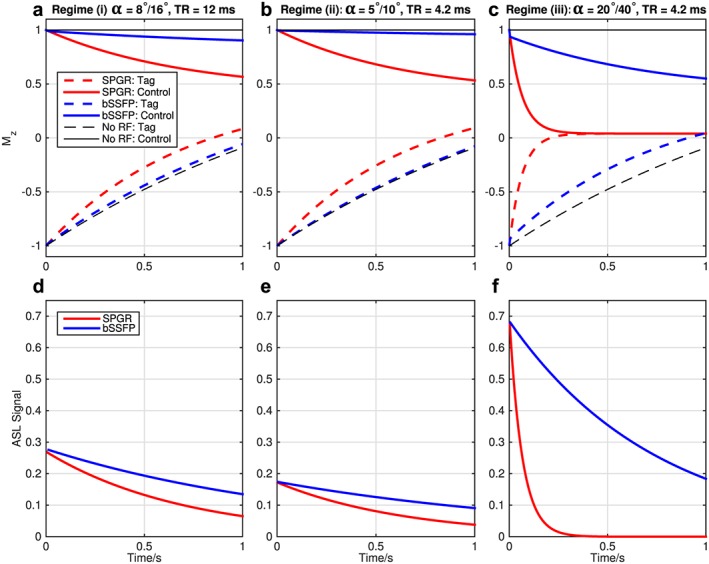
Motivation for a bSSFP readout in ASL angiography: Bloch simulations of the blood magnetization during SPGR and bSSFP readouts in both tag (dashed lines) and control (solid lines) conditions. Here the half‐angle method was used to catalyze the steady state in bSSFP. The top row (a–c) shows the evolution of the longitudinal magnetization (*M_z_*, in units of *M*_0_) under both readout schemes and in the absence of readout RF pulses, for comparison. The bottom row (d–f) shows the ASL signal, calculated as the difference in the transverse magnetization between control and tag states at the echo time, in units of *M*_0_. Note that the bSSFP flip angle was set to twice the SPGR flip angle shown to match the ASL signal at early time points. Three regimes are shown: (i) a relatively small flip angle (*α*, stated for SPGR/bSSFP) and long *T*_R_ (a, d); (ii) a short *T*_R_ and lower flip angle to give more rapid *k*‐space sampling with a similar degree of signal attenuation (b, e); (iii) a more demanding case, using short *T*_R_ with a higher flip angle to increase the initial SNR (c, f). bSSFP allows high ASL signals to be maintained over a much longer period of time than SPGR, even when the flip angle is large and the *T*_R_ is short, suggesting that significant boosts in SNR efficiency should be achievable.

Simulation results comparing the evolution of the longitudinal magnetization and the resulting ASL signal for both SPGR and bSSFP are shown in Figure 2. The recycling of the transverse magnetization across *T*_R_ periods in bSSFP results in greatly reduced attenuation of the longitudinal magnetization compared with SPGR, yielding higher ASL signals at later time points. Reasonable signal levels are achieved with SPGR in Regime (i), but the relatively long *T*_R_ leads to long acquisition times, making it unfeasible for use in a 4D protocol. Reducing the *T*_R_ to allow more rapid sampling requires a corresponding reduction in the flip angle to achieve the same degree of signal attenuation (Regime (ii)), thereby reducing the signal level considerably. Attempting to recover some of this SNR by increasing the flip angle (Regime (iii)) leads to rapid SPGR signal loss. In contrast, the bSSFP imaging sequence maintains high signal levels across all time points, even when the *T*_R_ is short and the flip angle is large.

**Figure 3:**
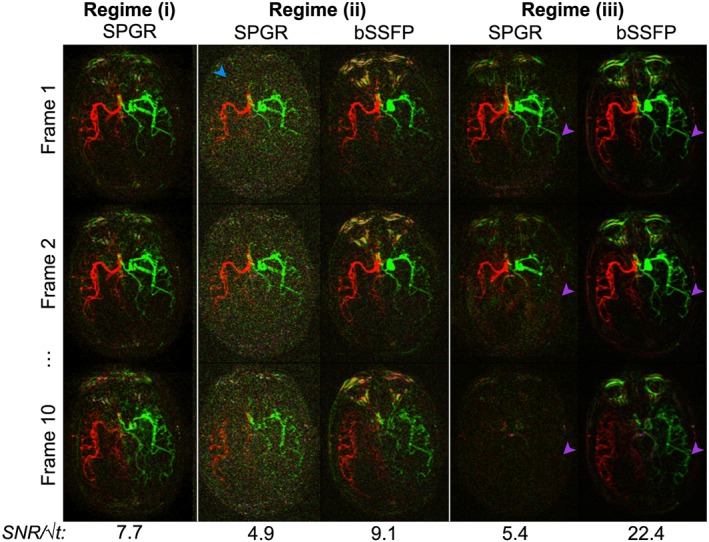
Selected frames from an experimental comparison of bSSFP and SPGR in the same subject using dynamic 2D mode without inflow subtraction. Three regimes are shown here, matching the simulations shown in Figure 2, with flip angle/*T*_R_/acquisition time approximately equal to (i) 8°/12 ms/5 min, (ii) 5°/4 ms/1.5 min, and (iii) 20°/4 ms/1.5 min. Note that bSSFP data were acquired with twice the SPGR flip angle (as per Fig. 2) but cannot be acquired in Regime (i) because the long *T*_R_ would lead to significant banding artifacts. SNR efficiencies are displayed below each set of images. Good quality SPGR data can be acquired in Regime (i), but accelerating the acquisition by reducing the *T*_R_ and the flip angle leads to poor SNR (blue arrowhead) in Regime (ii). bSSFP data acquired in Regime (ii) are comparable to those of SPGR in terms of signal attenuation, but because no spoiler is required at the end of each *T*_R_ period a lower bandwidth could be used, yielding higher SNR images. Attempting to recover some SNR by increasing the flip angle in Regime (iii) leads to rapid signal loss for SPGR, but not bSSFP (purple arrowheads). Note that the signal loss in proximal vessels is less severe due to the inflow of fresh labeled blood, which has not experienced all the previous RF excitation pulses.

This behavior is confirmed by the experimental comparison of bSSFP and SPGR in dynamic 2D transverse acquisitions (Fig. 3). The measured SNR efficiency of bSSFP in Regime (iii) is 2.9 times higher than the best SPGR protocol (Regime (i)). These results highlight that a much higher SNR and *k*‐space sampling rate can be achieved with bSSFP compared with SPGR, improving image quality and sampling efficiency, and thereby enabling the use of a time‐resolved 3D readout.

**Figure 4:**
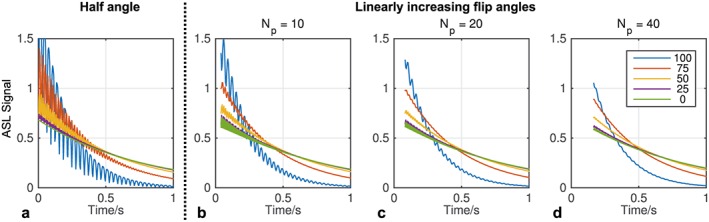
Minimizing transient signal oscillations: the ASL signal (in units of *M*_0_) produced at different off‐resonance frequencies (see legend, in Hz) was simulated during the bSSFP readout using the half‐angle (a) and linearly increasing flip angle (b–d) methods (*α* = 40°, *T*_R_ = 4.2 ms). The half‐angle method effectively suppresses signal oscillations on resonance but fails as the off‐resonance frequency is increased. Using a larger number of pre‐pulses (*N*_p_) with linearly increasing flip angles effectively suppresses these oscillations across a range of off‐resonance frequencies (c, d) at a cost of decreased time available for imaging immediately after the ASL preparation.

Simulations of off‐resonance magnetization (Fig. 4) show that the ASL difference signal is strongly affected by transient signal oscillations when the half‐angle catalyzation method is used. Using a set of linearly increasing flip angles greatly reduces these oscillations across a range of off‐resonance frequencies, with a larger number of pre‐pulses (*N*_p_) giving more effective damping. However, this is at a cost of reduced time available for imaging just after the VEPCASL pulse train, when the signal is strongest. Based on these simulations *N*_p_ was set to 20 for the remainder of this study as a reasonable compromise between these two competing factors. Note that despite the use of this catalyzation scheme some small transient oscillations remain, particularly at early time points, which could lead to minor artifacts in the resulting images.

**Figure 5:**
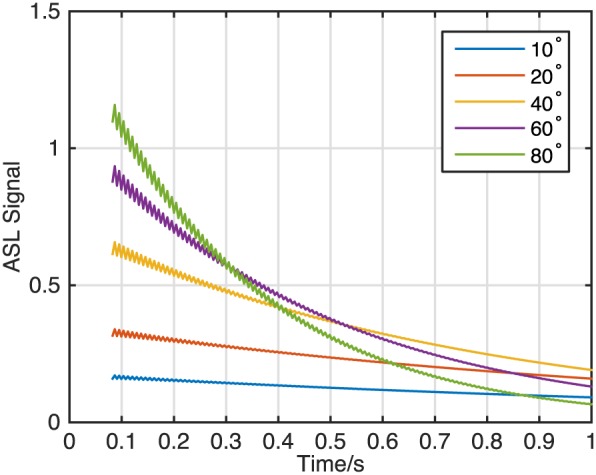
bSSFP readout flip angle optimization: simulations show that larger flip angles (see legend) provide higher SNR at early time points but attenuate the ASL signal more rapidly, leaving lower signals at later time points. For this simulation the magnetization was on‐resonance, *T*_R_ was set to 4.2 ms, and 20 linearly increasing flip angle pulses were used to reduce transient oscillations.

The simulations also demonstrate that increasing the bSSFP readout flip angle yields higher initial signals at a cost of greater signal attenuation at later time points (Fig. 5). For the purposes of this study, with a desired readout period of under 1 s, setting the flip angle to 40° yields the highest signal at later time points without compromising too greatly on initial signal amplitude, so this value was chosen for the experiments described below.

**Figure 6:**
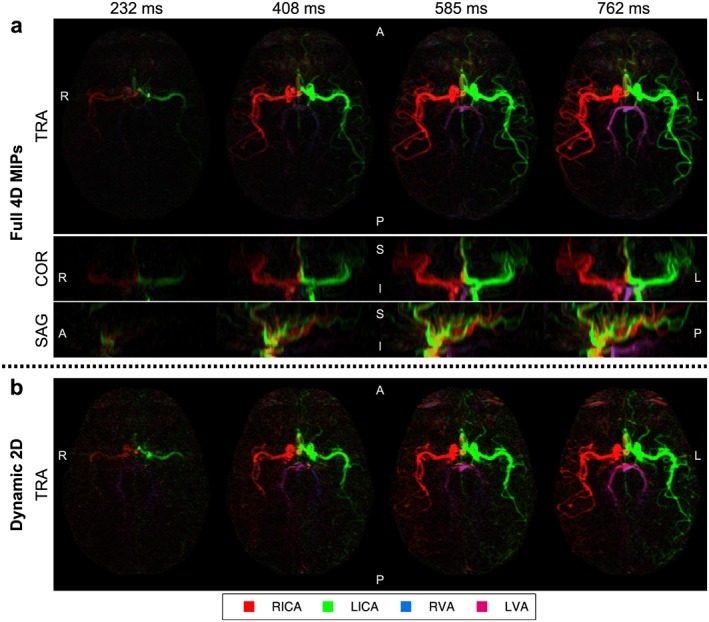
Selected frames from bSSFP VEPCASL dynamic angiography data in a single subject with inflow‐subtraction: the full 4D data (a) are shown using MIPs in transverse (TRA), coronal (COR) and sagittal (SAG) orientations. Transverse dynamic 2D data acquired in the same imaging slab are also shown (b). Color corresponds to the arterial origin of the blood signal (see legend). Note that overlap of the RICA and LICA signals in the sagittal MIP presents as a yellow hue. The times shown are relative to the end of the VEPCASL pulse train. Orientation is marked on each image (A, anterior; P, posterior; R, right; L, left; S, superior; I, inferior).

Selected frames from bSSFP 4D and dynamic 2D VEPCASL angiograms acquired in the same subject with inflow‐subtraction are shown in Figure 6. The 4D data are visualized using MIPs in transverse, coronal and sagittal views, although any desired reformatting could be chosen retrospectively. The expected patterns of filling of the major arteries are observed in this dynamic data and the vascular territories of the major brain‐feeding arteries are cleanly separated. The transverse dynamic 2D data acquired in the same imaging slab closely resemble the 4D transverse MIP despite the greatly reduced imaging time (~1.5 min).

**Figure 7:**
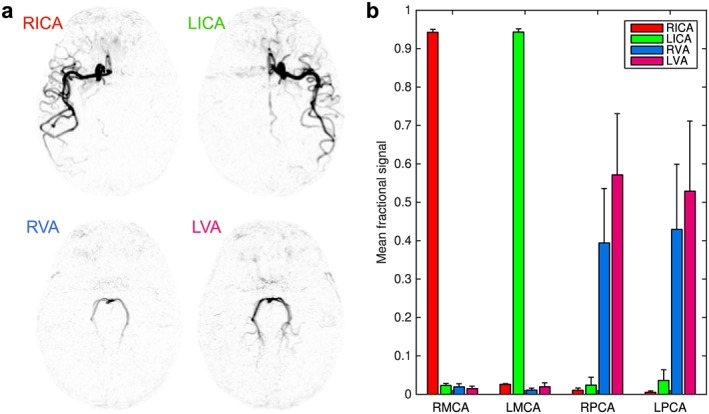
Vessel‐selectivity: (a) inverted grayscale maps from one frame of the transverse MIP shown in Figure 6, showing each arterial component separately, demonstrating the excellent separation of vascular components achieved with this technique; (b) mean fractional signal intensity from each feeding artery in ROIs placed over the MCAs and PCAs across all subjects. Error bars indicate the standard error. Color shows the origin of the blood signal (see legend). Note the minimal contamination signal from the non‐feeding arteries.

The high vessel selectivity achievable with this vessel‐encoded approach is demonstrated in Figure 7, where each arterial component is displayed separately. Analysis of the signals within the MCA and PCA ROIs confirms that the signal contamination is very low: the average signal from non‐feeding arteries was only 1.9 ± 2.0%.

Examples of timing information extracted from this data are shown in Supplementary Fig. 1. The later arrival of blood in more distal arteries, as well as the delayed arrival of blood to the posterior circulation versus the anterior circulation, is clearly shown, and the timing information is very similar between the 4D and dynamic 2D data sets. High quality data sets were obtained in all subjects, as shown in Supplementary Fig. 2, despite some minor artifacts related to shifting bSSFP banding patterns and motion in some subjects. This technique clearly shows variants of the cerebral vasculature such as a fetal type circle of Willis.

The qualitative comparison of 3D TOF and 4D bSSFP VEPCASL angiography revealed no significant differences in vessel visualization between the two methods (see Supplementary Table 2). On average, the proposed method scored slightly lower in proximal vessels due to the slightly lower spatial resolution used in this study and minor artifacts seen in some subjects, but in distal MCA branches arterial visualization was much better (mean score 2.6 for VEPCASL versus 1.1 for TOF), with a trend towards significance (*p* = 0.06).

**Figure 8:**
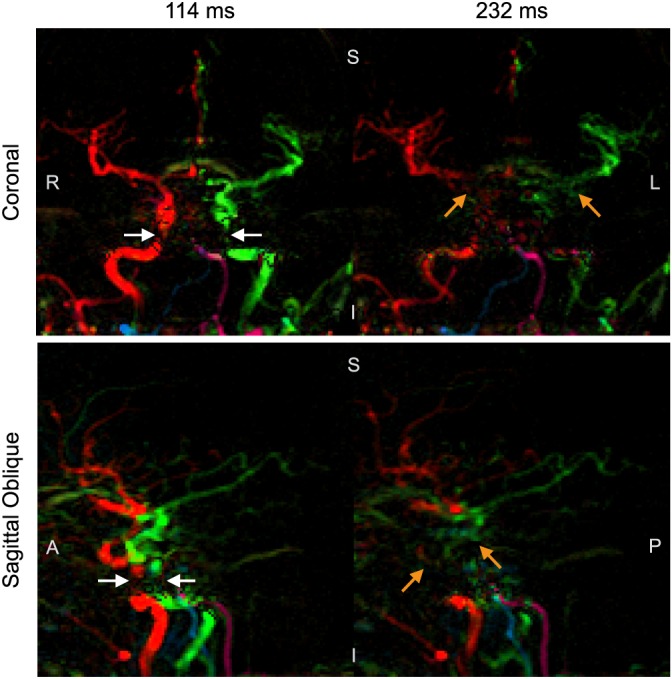
Signal loss in proximal vessels during coronal and sagittal acquisitions: two frames from dynamic 2D data without inflow‐subtraction showing loss of signal in proximal arteries (white arrows), which propagates through to more distal regions in later time frames (orange arrows) despite the presence of labeled blood signal upstream. The time of image acquisition relative to the end of the VEPCASL pulse train is displayed above each frame. Subject orientation is indicated on each image (R, right; L, left; S, superior; I, inferior; A, anterior; P, posterior).

One drawback of using bSSFP for ASL angiography is highlighted in Figure 8, which shows example results from dynamic 2D acquisitions in coronal and sagittal views. Regions with poor magnetic field homogeneity show signal loss in the first time frame, which rapidly propagates downstream. In this manner large sections of arteries lose all signal in later time frames, despite the presence of labeled blood in proximal vessel segments.

## Discussion

In this study we have demonstrated that for dynamic ASL angiography bSSFP can provide a much higher SNR efficiency than SPGR without significantly attenuating the ASL signal, we have optimized bSSFP imaging parameters for a relatively long readout period, and we have combined this optimized bSSFP readout with vessel‐encoded ASL to produce 4D vessel‐selective cerebral angiograms non‐invasively with high spatial and temporal resolution. These 4D angiograms can be reconstructed using MIPs in any desired orientation, and the vessel selectivity was shown to be high. In addition, the dynamic 2D protocol allows for a very rapid acquisition (~1.5 min), which could fit easily into a busy clinical protocol.

The simulations showed that transient signal oscillations were well suppressed using 20 RF pulses with linearly increasing flip angles, although this uses up 84 ms of potential imaging time just after the end of the VEPCASL pulse train. For this study a flip angle of 40° was found to provide a good compromise between initial signal strength and the rate of signal attenuation. This is somewhat different from the values used in previous studies 17, 19, where flip angles of up to 180° have been used 26. This is due to the optimization being performed over a relatively long readout period (~700 ms) and considering blood that has experienced all previous RF pulses, which may not be the case in proximal vessels. This highlights the need for optimization to be performed based on the desired readout duration and vessels of interest within the imaging region.

The images produced using this technique in healthy volunteers confirm that bSSFP is capable of producing high quality angiograms when combined with a VEPCASL preparation. Even relatively small arterial branches are clearly visualized thanks to the high SNR. These details are less well defined in the dynamic 2D acquisitions, but this is perhaps not surprising given the much shorter imaging time. The ability of this method to clearly visualize normal variants of the cerebral vasculature suggests that it could be useful in identifying abnormal flow patterns in a range of patient groups, including collateral flow and blood supply to lesions such as tumors or arteriovenous malformations.

Although the post‐processing is more complex, the combination of a vessel‐encoded preparation and Bayesian analysis method 38, 39 has two main advantages over single‐artery labeling methods 26, 42, 43, 44 for vessel‐selective angiography. First, it is very SNR efficient when more than one artery is of interest 27, as described in the introduction. Second, it yields highly vessel‐selective angiograms: the average signal contamination in this study was 1.9 ± 2.0%, which is considerably lower than the average value of 12 ± 7% reported by Robson *et al*. 26 using single‐artery selective PCASL. This has the advantage of reducing ambiguity in the resulting images: for example, signal present in vessels not normally supplied by the labeled artery can be more confidently ascribed to collateral flow rather than signal contamination. This finding needs to be reproduced in patient groups where motion may be more pronounced, although one could argue that single‐artery selective methods are even more susceptible to motion since mislabeling of arteries cannot be corrected for in post‐processing.

The outflow timing parameter extracted from these data could be used to help identify abnormal hemodynamics, such as delayed flow due to stenosis or increased vascular resistance. However, this simple metric is affected by signal attenuation from the readout method and bolus dispersion. To more accurately extract this information, a kinetic model for ASL angiography 15 needs to be adapted to account for the signal attenuation present in bSSFP acquisitions.

Unlike previous studies 10, here only the magnitude images were used for the analysis, rather than the full complex data set. This was possible because the phase of the bSSFP signal is relatively insensitive to off‐resonance frequency and flow speed 45. Therefore, phase mismatches between static tissue and blood, which could cause problems in SPGR magnitude subtractions, are not generally an issue for bSSFP. The use of magnitude‐only images simplifies the post‐processing, since no phase drift corrections are required and problems with phase instabilities 10 are avoided. However, near the labeling plane, where the longitudinal magnetization of the blood may still be negative at the time of imaging, the ASL signal will be underestimated. Therefore, full complex processing is necessary when quantitative analyses are to be performed.

The inflow‐subtraction approach used in this study is a useful visualization tool, but it does make the assumption that all the vessels are filled with labeled blood at the first time point. This assumption would be violated for delayed blood arrival, so these images should be viewed alongside the original data to avoid potential misinterpretation. This method also assumes that the blood signal is constant until the bolus of labeled blood washes out of the voxel, but the application of the RF pulses for imaging attenuates the signal over time (see Fig. 2), which would lead to a slowly rising signal over time in the inflow‐subtraction before the nominal “arrival” of the bolus. Finally, the SNR of these images is reduced due to increased noise variance resulting from the subtraction. One possible solution to these problems would be to adapt the PCASL angiography kinetic model to account for the bSSFP signal evolution, allowing generation of simulated inflow images without the confounds of signal attenuation due to the imaging RF pulses and *T*_1_ decay, as has been show previously using SPGR data 15. This would further allow physiological parameters to be extracted and absolute blood volume flow rates to be estimated 46.

The qualitative comparison of the proposed technique with 3D TOF revealed that comparable structural information is obtained with the two methods, in addition to the dynamic and vessel‐selective nature of the 4D bSSFP VEPCASL data. It is hoped that the bSSFP VEPCASL angiography scores in proximal vessels, particular smaller vessels such as the ACAs, could be improved by using a higher spatial resolution. The trend towards higher scores in distal MCA branches is likely to be due to the non‐negligible static tissue signal and saturation effects inherent in the 3D TOF data, suggesting that ASL angiography is particularly advantageous higher in the brain. However, the 3D TOF protocol used in this study was suboptimal, the scan times were not matched, and a relatively small number of subjects were included, so a larger study would be required to investigate these differences further.

In previous studies 47, 48 it has been shown that in many subjects there is little mixing of VA blood in the basilar artery. In this study we observed subjects with both relatively well‐separated and well‐mixed VA blood (see Fig. 6 and Supplementary Fig. 2). Such variable patterns have also previously been observed in vessel‐selective perfusion images, using both a vessel‐encoded approach 28 and a single‐artery selective approach 49. This highlights one of the benefits of the Bayesian analysis method used in this study over clustering methods, which is the ability to accurately depict mixed blood supply.

Although bSSFP has many advantages for ASL angiography, it also has some drawbacks. First, there are some residual transient bSSFP oscillation artifacts in the first acquired time frame. Although these are small, they necessitated the use of the second time frame for inflow‐subtraction to avoid these artifacts propagating through the whole set of images. More sophisticated methods for suppressing these oscillations 50, 51 may be beneficial in future work. Second, bSSFP may be more sensitive than SPGR to fluctuations in blood flow and cerebrospinal fluid during the cardiac cycle 52, prompting the use of cardiac gating, which can lead to dead time in the sequence whilst waiting for the next trigger. However, in this study the number of readout blocks was adapted for each subject, ensuring that the VEPCASL preparation and imaging period were complete within two cardiac cycles to minimize this dead time. It should be noted that, in patients with highly variable heart rates, *k*‐space data within the same readout block could be acquired at different points during the cardiac cycle, so assessment of pulsatility artifacts in such cohorts would be necessary. Third, drift in the main magnetic field (e.g. due to heating of the passive shim metal) causes the bSSFP banding artifacts to move. During the longer 4D protocol this led to minor artifacts in the ASL images in some subjects.

Finally, it appears that the bSSFP readout leads to rapid ASL signal loss in proximal areas when the imaging region extends into the neck (see Fig. 8). This is most likely caused by off‐resonance effects in these larger and less homogeneous imaging volumes that are difficult to shim accurately. It has been shown previously that motion of fluid through regions of poor field homogeneity can disrupt the evolution of the magnetization under the bSSFP imaging scheme, leading to considerable signal loss 45. This is particularly problematic for ASL angiography, because it is not a localized effect: saturated blood signal flows downstream, leading to signal loss in distal arteries at later time points. Note that lack of flow compensation in the phase‐encoding direction could also contribute to the signal loss seen in the fast moving blood found in these proximal arteries 29. However, previous studies have demonstrated that bSSFP can be used to image the extracranial arteries at 1.5 T 24, perhaps because improved field homogeneity at this lower field strength and the short *T*_R_ period used (3.4 ms) mitigate bSSFP off‐resonance effects. Further investigation of these artifacts is necessary before simultaneous examination of the intracranial and extracranial vessels becomes feasible at 3 T.

The 4D VEPCASL angiography protocol presented here took 18 min and could therefore be sensitive to subject motion and magnetic field drift. Reducing the acquisition time would help reduce this sensitivity as well as improving its clinical utility. This could be achieved in a number of ways: the temporal resolution could be increased to allow more lines of *k*‐space to be acquired after each VEPCASL preparation. The number of vessel encodings could be reduced from eight to six 10 or even five, with a planning‐free approach 53, although SNR and robust vessel‐decoding could be somewhat compromised. Greater acceleration with parallel imaging 54, 55 should be possible, particularly using a receive coil with a larger number of elements, although again with an SNR penalty. Compressed sensing 56 and radial *k*‐space trajectories 41 are also likely to yield great benefits due to the inherently sparse nature of angiographic data.

This work could be further improved by more efficient suppression of static tissue signals, for example by interleaving inversion pulses with the VEPCASL pulse train 26, helping to reduce motion artifacts and reduce physiological noise. Use of a real‐time magnetic field drift correction would also eliminate the motion of bSSFP banding artifacts.

Finally, the use of variable flip angles in the bSSFP readout has been shown to be beneficial in pulsed ASL angiography 21. Optimization of such a scheme for PCASL, which has different *T*_1_ decay properties, is likely to be beneficial, and there are a number of approaches for achieving this 51, 57.

In conclusion, in this study we have demonstrated that bSSFP has many advantages over SPGR for intracranial ASL dynamic angiography. When combined with a VEPCASL preparation it can produce very SNR‐efficient vessel‐selective 4D angiograms with high spatial and temporal resolution and very rapid dynamic 2D data sets suitable for busy clinical protocols. In future work we hope to exploit the short time required per vessel‐encoding cycle to label a larger number of arterial branches higher in the brain, and to show the efficacy of this technique in a range of clinical scenarios, such as identifying dominant feeding arteries to arteriovenous malformations and assessing collateral flow in stroke patients.

## Supporting information


**Supplementary Table 1:** Sequence parameters for the bSSFP vs. SPGR comparison in one subject using dynamic 2D mode. Parameters not listed here were identical to those in Table 1. The readout bandwidth was set to the minimum possible within the set TR period. Note that an exact match between SPGR and bSSFP protocols could not always be made due to hardware restrictions.
**Supplementary Table 2:** Qualitative image quality scores comparing 3D TOF and 4D VEPCASL angiography. Mean ± standard deviation scores are shown for each vessel segment, along with the p value resulting from the paired Wilcoxon signed rank test for that vessel segment. No differences were statistically significant (p < 0.05).
**Supplementary Fig. 1:** Comparison of timing information obtained by the full 4D (a) and dynamic 2D (b) methods using a simple outflow metric.
**Supplementary Fig. 2:** One frame from transverse MIPs of the 4D data in the other four subjects (a‐d), two of which show clear visualization of variants in the cerebral vasculature: one subject (a) has a very small RVA which does not supply blood to the circle of Willis; another subject (c) has a very small LVA and fetal type circle of Willis on the left side. Note that shifting of bSSFP banding artifacts (white arrowhead) and subject motion (orange arrowhead) lead to minor artifacts in some subjects.

Supporting info item

Supporting info item

Supporting info item

## References

[1] Liebeskind DS . Collateral circulation. Stroke 2003; 34: 2279–2284. 12881609 10.1161/01.STR.0000086465.41263.06

[2] Kaufmann TJ , Huston J, 3rd , Mandrekar JN , Schleck CD , Thielen KR , Kallmes DF . Complications of diagnostic cerebral angiography: evaluation of 19,826 consecutive patients. Radiology 2007; 243: 812–819. 17517935 10.1148/radiol.2433060536

[3] Williams DS , Detre JA , Leigh JS , Koretsky AP . Magnetic resonance imaging of perfusion using spin inversion of arterial water. Proc. Natl. Acad. Sci. U. S. A. 1992; 89: 212–216. 1729691 10.1073/pnas.89.1.212 PMC48206

[4] Detre JA , Leigh JS , Williams DS , Koretsky AP . Perfusion imaging. Magn. Reson. Med. 1992; 23: 37–45. 1734182 10.1002/mrm.1910230106

[5] Dixon WT , Du LN , Faul DD , Gado M , Rossnick S . Projection angiograms of blood labeled by adiabatic fast passage. Magn. Reson. Med. 1986; 3: 454–462. 3724425 10.1002/mrm.1910030311

[6] Edelman RR , Siewert B , Adamis M , Gaa J , Laub G , Wielopolski P . Signal targeting with alternating radiofrequency (STAR) sequences: application to MR angiography. Magn. Reson. Med. 1994; 31: 233–238. 8133761 10.1002/mrm.1910310219

[7] Nishimura DG , Macovski A , Pauly JM , Conolly SM . MR angiography by selective inversion recovery. Magn. Reson. Med. 1987; 4: 193–202. 3561250 10.1002/mrm.1910040214

[8] Wang SJ , Nishimura DG , Macovski A . Multiple‐readout selective inversion recovery angiography. Magn. Reson. Med. 1991; 17: 244–251. 2067399 10.1002/mrm.1910170127

[9] Sallustio F , Kern R , Günther M , Szabo K , Griebe M , Meairs S , Hennerici M , Gass A . Assessment of intracranial collateral flow by using dynamic arterial spin labeling MRA and transcranial color‐coded duplex ultrasound. Stroke 2008; 39: 1894–1897. 18403739 10.1161/STROKEAHA.107.503482

[10] Okell TW , Chappell MA , Woolrich MW , Günther M , Feinberg DA , Jezzard P . Vessel‐encoded dynamic magnetic resonance angiography using arterial spin labeling. Magn. Reson. Med. 2010; 64: 698–706. 20535814 10.1002/mrm.22458

[11] Kopeinigg D , Bammer R . Time‐resolved angiography using inflow subtraction (TRAILS). Magn. Reson. Med. 2014; 72: 669–678. 24166577 10.1002/mrm.24985 PMC4396866

[12] Nakamura M , Yoneyama M , Tabuchi T , Takemura A , Obara M , Tatsuno S , Sawano S . Vessel‐selective, non‐contrast enhanced, time‐resolved MR angiography with vessel‐selective arterial spin labeling technique (CINEMA‐SELECT) in intracranial arteries. Radiol. Phys. Technol. 2013; 6: 327–334. 23475783 10.1007/s12194-013-0204-7

[13] Wu H , Block WF , Turski PA , Mistretta CA , Rusinak DJ , Wu Y , Johnson KM . Noncontrast dynamic 3D intracranial MR angiography using pseudo‐continuous arterial spin labeling (PCASL) and accelerated 3D radial acquisition. J. Magn. Reson. Imaging 2014; 39: 1320–1326. 24129947 10.1002/jmri.24279 PMC3984365

[14] Lindner T , Jensen‐Kondering U , van Osch MJP , Jansen O , Helle M . 3D time‐resolved vessel‐selective angiography based on pseudo‐continuous arterial spin labeling. Magn. Reson. Imaging 2015; 33: 840–846. 25777269 10.1016/j.mri.2015.03.001

[15] Okell TW , Chappell MA , Schulz UG , Jezzard P . A kinetic model for vessel‐encoded dynamic angiography with arterial spin labeling. Magn. Reson. Med. 2012; 68: 969–979. 22246669 10.1002/mrm.23311 PMC6783303

[16] Scheffler K , Lehnhardt S . Principles and applications of balanced SSFP techniques. Eur. Radiol. 2003; 13: 2409–2418. 12928954 10.1007/s00330-003-1957-x

[17] Martirosian P , Klose U , Mader I , Schick F . FAIR true‐FISP perfusion imaging of the kidneys. Magn. Reson. Med. 2004; 51: 353–361. 14755661 10.1002/mrm.10709

[18] Boss A , Martirosian P , Klose U , Nägele T , Claussen CD , Schick F . FAIR‐TrueFISP imaging of cerebral perfusion in areas of high magnetic susceptibility differences at 1.5 and 3 Tesla. J. Magn. Reson. Imaging 2007; 25: 924–931. 17410577 10.1002/jmri.20893

[19] Yan L , Wang S , Zhuo Y , Wolf RL , Stiefel MF , An J , Ye Y , Zhang Q , Melhem ER , Wang DJJ . Unenhanced dynamic MR angiography: high spatial and temporal resolution by using true FISP‐based spin tagging with alternating radiofrequency. Radiology 2010; 256: 270–279. 20574100 10.1148/radiol.10091543 PMC2897689

[20] Bi X , Weale P , Schmitt P , Zuehlsdorff S , Jerecic R . Non‐contrast‐enhanced four‐dimensional (4D) intracranial MR angiography: a feasibility study. Magn. Reson. Med. 2010; 63: 835–841. 20187191 10.1002/mrm.22220

[21] Schmitt P , Speier P , Bi X , Weale P , Mueller E . Non‐contrast‐enhanced 4D intracranial MR angiography: optimizations using a variable flip angle approach. In *Proceedings 18th Scientific Meeting*, *ISMRM*, Stockholm, 2010 402.

[22] Song HK , Yan L , Smith RX , Xue Y , Rapacchi S , Srinivasan S , Ennis DB , Hu P , Pouratian N , Wang DJJ . Noncontrast enhanced four‐dimensional dynamic MRA with golden angle radial acquisition and K‐space weighted image contrast (KWIC) reconstruction. Magn. Reson. Med. 2014; 72: 1541–1551. 24338944 10.1002/mrm.25057 PMC4055554

[23] Yan L , Salamon N , Wang DJJ . Time‐resolved noncontrast enhanced 4‐D dynamic magnetic resonance angiography using multibolus TrueFISP‐based spin tagging with alternating radiofrequency (TrueSTAR). Magn. Reson. Med. 2014; 71: 551–560. 23440649 10.1002/mrm.24689 PMC3675183

[24] Koktzoglou I , Meyer JR , Ankenbrandt WJ , Giri S , Piccini D , Zenge MO , Flanagan O , Desai T , Gupta N , Edelman RR . Nonenhanced arterial spin labeled carotid MR angiography using three‐dimensional radial balanced steady‐state free precession imaging. J. Magn. Reson. Imaging 2015; 41: 1150–1156. 24737420 10.1002/jmri.24640

[25] van Osch MJ , Hendrikse J , Golay X , Bakker CJ , van der Grond J . Non‐invasive visualization of collateral blood flow patterns of the circle of Willis by dynamic MR angiography. Med. Image Anal. 2006; 10: 59–70. 15950521 10.1016/j.media.2005.04.010

[26] Robson PM , Dai W , Shankaranarayanan A , Rofsky NM , Alsop DC . Time‐resolved vessel‐selective digital subtraction MR angiography of the cerebral vasculature with arterial spin labeling. Radiology 2010; 257: 507–515. 20959548 10.1148/radiol.10092333 PMC2957593

[27] Wong EC . Vessel‐encoded arterial spin‐labeling using pseudocontinuous tagging. Magn. Reson. Med. 2007; 58: 1086–1091. 17969084 10.1002/mrm.21293

[28] Okell TW , Chappell MA , Kelly ME , Jezzard P . Cerebral blood flow quantification using vessel‐encoded arterial spin labeling. J. Cereb. Blood Flow Metab. 2013; 33: 1716–1724. 23921895 10.1038/jcbfm.2013.129 PMC3824178

[29] Bieri O , Scheffler K . Flow compensation in balanced SSFP sequences. Magn. Reson. Med. 2005; 54: 901–907. 16142709 10.1002/mrm.20619

[30] Alsop DC , Detre JA , Golay X , Günther M , Hendrikse J , Hernandez‐Garcia L , Lu H , MacIntosh BJ , Parkes LM , Smits M , van Osch MJP , Wang DJJ , Wong EC , Zaharchuk G . Recommended implementation of arterial spin‐labeled perfusion MRI for clinical applications: a consensus of the ISMRM Perfusion Study Group and the European consortium for ASL in Dementia. Magn. Reson. Med. 2015; 73: 102–116. 24715426 10.1002/mrm.25197 PMC4190138

[31] Lu H , Xu F , Grgac K , Liu P , Qin Q , van Zijl P . Calibration and validation of TRUST MRI for the estimation of cerebral blood oxygenation. Magn. Reson. Med. 2012; 67: 42–49. 21590721 10.1002/mrm.22970 PMC3158970

[32] Deimling M , Heid O . Magnetization prepared true FISP imaging. In *Proceedings Second Annual Meeting*, *Society for Magnetic Resonance*, San Francisco, CA, 1994 495.

[33] Nishimura DG , Vasanawala SS . Analysis and reduction of the transient response in SSFP imaging. *Proceedings Eighth Scientific Meeting*, *ISMRM*, Denver, CO, 2000 301.

[34] Jenkinson M , Beckmann CF , Behrens TE , Woolrich MW , Smith SM . FSL. NeuroImage 2012; 62: 782–790. 21979382 10.1016/j.neuroimage.2011.09.015

[35] Jenkinson M , Bannister P , Brady M , Smith S . Improved optimization for the robust and accurate linear registration and motion correction of brain images. Neuroimage 2002; 17: 825–841. 12377157 10.1016/s1053-8119(02)91132-8

[36] Smith SM . Fast robust automated brain extraction. Hum. Brain Mapp. 2002; 17: 143–155. 12391568 10.1002/hbm.10062 PMC6871816

[37] Jenkinson M , Smith S . A global optimisation method for robust affine registration of brain images. Med. Image Anal. 2001; 5: 143–156. 11516708 10.1016/s1361-8415(01)00036-6

[38] Chappell MA , Okell TW , Payne SJ , Jezzard P , Woolrich MW . A fast analysis method for non‐invasive imaging of blood flow in individual cerebral arteries using vessel‐encoded arterial spin labelling angiography. Med. Image Anal. 2012; 16: 831–839. 22322066 10.1016/j.media.2011.12.004 PMC3398734

[39] Chappell MA , Okell TW , Jezzard P , Woolrich MW . A general framework for the analysis of vessel encoded arterial spin labeling for vascular territory mapping. Magn. Reson. Med. 2010; 64: 1529–1539. 20677231 10.1002/mrm.22524

[40] Okell TW , Schmitt P , Bi X , Chappell MA , Tijssen RH , Miller KL , Jezzard P . 4D vessel‐encoded arterial spin labeling angiography. In *Proceedings 19th Scientific Meeting*, *ISMRM*, Montreal, 2011 4034.

[41] Wu H , Block WF , Turski PA , Mistretta CA , Johnson KM . Noncontrast‐enhanced three‐dimensional (3D) intracranial MR angiography using pseudocontinuous arterial spin labeling and accelerated 3D radial acquisition. Magn. Reson. Med. 2013; 69: 708–715. 22532423 10.1002/mrm.24298 PMC3424331

[42] Dai W , Robson PM , Shankaranarayanan A , Alsop DC . Modified pulsed continuous arterial spin labeling for labeling of a single artery. Magn. Reson. Med. 2010; 64: 975–982. 20665896 10.1002/mrm.22363 PMC3266713

[43] Helle M , Norris DG , Rüfer S , Alfke K , Jansen O , van Osch MJP . Superselective pseudocontinuous arterial spin labeling. Magn. Reson. Med. 2010; 64: 777–786. 20597127 10.1002/mrm.22451

[44] Helle M , Rüfer S , Alfke K , Jansen O , Norris DG . Perfusion territory imaging of intracranial branching arteries – optimization of continuous artery‐selective spin labeling (CASSL). NMR Biomed. 2011; 24: 404–412. 22945292 10.1002/nbm.1605

[45] Storey P , Li W , Chen Q , Edelman RR . Flow artifacts in steady‐state free precession cine imaging. Magn. Reson. Med. 2004; 51: 115–122. 14705051 10.1002/mrm.10665

[46] Okell TW , Chappell MA , Jezzard P . A theoretical framework for quantifying blood volume flow rate from dynamic angiographic data and application to vessel‐encoded arterial spin labeling MRI. Med. Image Anal. 2013; 17: 1025–1036. 23871963 10.1016/j.media.2013.06.005 PMC3898265

[47] Kansagra AP , Wong EC . Mapping of vertebral artery perfusion territories using arterial spin labeling MRI. J. Magn. Reson. Imaging 2008; 28: 762–766. 18777538 10.1002/jmri.21462

[48] Bockman MD , Kansagra AP , Shadden SC , Wong EC , Marsden AL . Fluid mechanics of mixing in the vertebrobasilar system: comparison of simulation and MRI. Cardiovasc Eng. Technol. 2012; 3: 450–461.

[49] Hartkamp NS , Helle M , Chappell MA , Okell TW , Hendrikse J , Bokkers RPH , van Osch MJP . Validation of planning‐free vessel‐encoded pseudo‐continuous arterial spin labeling MR imaging as territorial‐ASL strategy by comparison to super‐selective p‐CASL MRI. Magn. Reson. Med. 2014; 71: 2059–2070. 23878062 10.1002/mrm.24872

[50] Le Roux P . Simplified model and stabilization of SSFP sequences. J. Magn. Reson. 2003; 163: 23–37. 12852904 10.1016/s1090-7807(03)00115-0

[51] Smith T , Zun Z , Wong EC , Nayak KS . Design and use of variable flip angle schedules in transient balanced SSFP subtractive imaging. Magn. Reson. Med. 2010; 63: 537–542. 20099336 10.1002/mrm.22255

[52] Tijssen RHN , Okell TW , Miller KL . Real‐time cardiac synchronization with fixed volume frame rate for reducing physiological instabilities in 3D FMRI. Neuroimage 2011; 57: 1364–1375. 21664465 10.1016/j.neuroimage.2011.05.070

[53] Gevers S , Bokkers RP , Hendrikse J , Majoie CB , Kies DA , Teeuwisse WM , Nederveen AJ , van Osch MJ . Robustness and reproducibility of flow territories defined by planning‐free vessel‐encoded pseudocontinuous arterial spin‐labeling. Am. J. Neuroradiol. 2012; 33: E21–25. 21393410 10.3174/ajnr.A2410 PMC7964817

[54] Pruessmann KP , Weiger M , Scheidegger MB , Boesiger P . SENSE: sensitivity encoding for fast MRI. Magn. Reson. Med. 1999; 42: 952–962. 10542355

[55] Griswold MA , Jakob PM , Heidemann RM , Nittka M , Jellus V , Wang J , Kiefer B , Haase A . Generalized autocalibrating partially parallel acquisitions (GRAPPA). Magn. Reson. Med. 2002; 47: 1202–1210. 12111967 10.1002/mrm.10171

[56] Lustig M , Donoho D , Pauly JM . Sparse MRI: the application of compressed sensing for rapid MR imaging. Magn. Reson. Med. 2007; 58: 1182–1195. 17969013 10.1002/mrm.21391

[57] Worters PW , Hargreaves BA . Balanced SSFP transient imaging using variable flip angles for a predefined signal profile. Magn. Reson. Med. 2010; 64: 1404–1412. 20632411 10.1002/mrm.22541 PMC2965793

