# Supplementary material for: Optimization of 4D vessel‐selective arterial spin labeling angiography using balanced steady‐state free precession and vessel‐encoding
Source: NMR Biomed. 2016 Apr 13;29(6):776–86. doi: 10.1002/nbm.3515 (PMC4879350; doi:10.1002/nbm.3515)
Supplement: Supplementary file 1 — Supporting info item [file NBM-29-776-s001.pdf]

**Sequence Parameter**	**Regime (i)**	**Regime (ii)**		**Regime (iii)**	
	SPGR	SPGR	bSSFP	SPGR	bSSFP
Repetition time (TR)/ms	12	4.5	4.2	4.5	4.2
Excitation flip angle/°	8	5	10	20	40
Echo time (TE)/ms	5.6	2.2	1.8	2.2	1.8
Readout bandwidth/Hz per pixel	130	797	496	797	496
Excitations per readout block	5	14	14	14	14
Temporal resolution/ms	60	63	59	63	59
Number of readout blocks	14	13	12	13	12
Total imaging time/min	5	1.5	1.5	1.5	1.5

Supplementary Table 1: Sequence parameters for the bSSFP vs. SPGR comparison in one subject using dynamic 2D mode. Parameters not listed here were identical to those in Table 1. The readout bandwidth was set to the minimum possible within the set TR period. Note that an exact match between SPGR and bSSFP protocols could not always be made due to hardware restrictions.

Vessel Segment	TOF Score	ASL Score	p
RACA1	2.8 ± 0.4	2.4 ± 0.9	0.750
RACA2	2.4 ± 0.5	1.6 ± 0.5	0.250
LACA1	2.2 ± 1.3	1.6 ± 1.5	0.500
LACA2	2.2 ± 0.4	1.6 ± 0.5	0.500
RMCA1	3.0 ± 0.0	2.2 ± 0.4	0.125
RMCA2	2.0 ± 0.0	2.2 ± 0.4	1.000
RMCA distal	1.0 ± 0.0	2.6 ± 0.5	0.062
LMCA1	3.0 ± 0.0	2.0 ± 0.7	0.125
LMCA2	2.0 ± 0.0	2.2 ± 0.4	1.000
LMCA distal	1.2 ± 0.4	2.6 ± 0.5	0.062
RPCA1	2.8 ± 0.4	2.4 ± 0.5	0.500
RPCA2	2.6 ± 0.5	2.2 ± 0.4	0.500
LPCA1	2.6 ± 0.5	2.4 ± 0.5	1.000
LPCA2	2.2 ± 0.4	2.2 ± 0.4	1.000

Supplementary Table 2: Qualitative image quality scores comparing 3D TOF and 4D VEPCASL angiography. Mean ± standard deviation scores are shown for each vessel segment, along with the p value resulting from the paired Wilcoxon signed rank test for that vessel segment. No differences were statistically significant ($p < 0.05$).
