# Supplementary figures and images for: Optimization of 4D vessel‐selective arterial spin labeling angiography using balanced steady‐state free precession and vessel‐encoding
Source: NMR Biomed. 2016 Apr 13;29(6):776–86. doi: 10.1002/nbm.3515 (PMC4879350; doi:10.1002/nbm.3515)

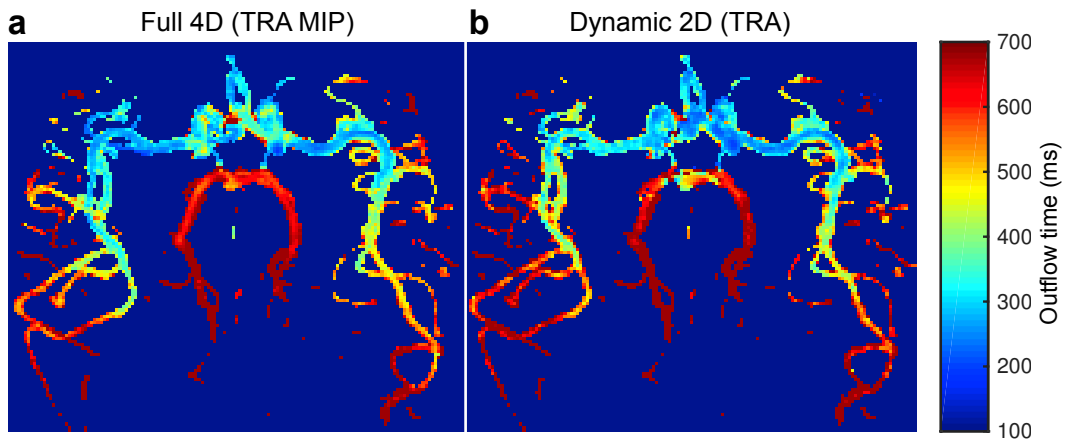

Supplement: Supplementary file 2 — Supporting info item [file NBM-29-776-s002.pdf]

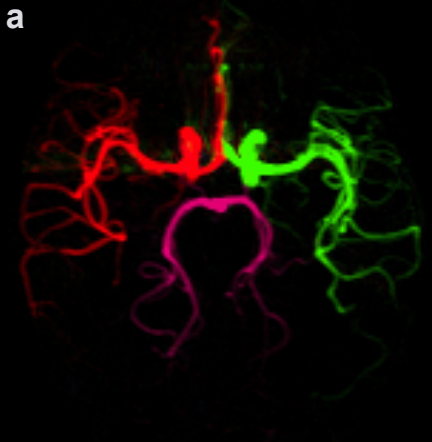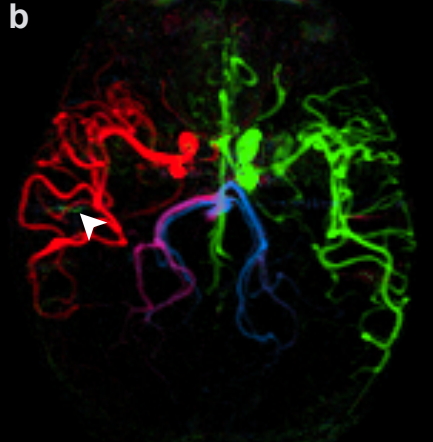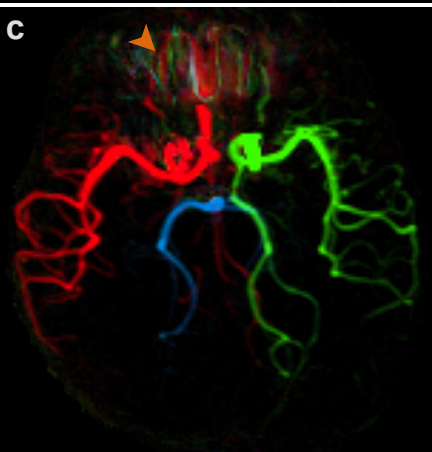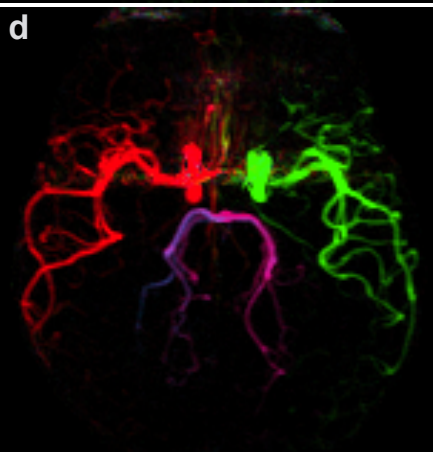

Supplement: Supplementary file 3 — Supporting info item [file NBM-29-776-s003.pdf]
